# Supplementary material for: Identification of photoperiod-induced specific miRNAs in the adrenal glands of Sunite sheep (Ovis aries)
Source: Front Vet Sci. 2022 Jul 22;9:888207. doi: 10.3389/fvets.2022.888207 (PMC9354845; doi:10.3389/fvets.2022.888207)
Supplement: Supplementary file 1 [file Data_Sheet_1.ZIP › Supplementary file/miRDeep2 file.zip!/miRDeep2 file/Know_Structure/SPLP42A1/oar-mir-154b.pdf]

miRBase precursor : oar-mir-154b
Total read count : 1709
oar-miR-154b-5p read count : 1610
oar-miR-154b-3p read count : 97
remaining reads : 2

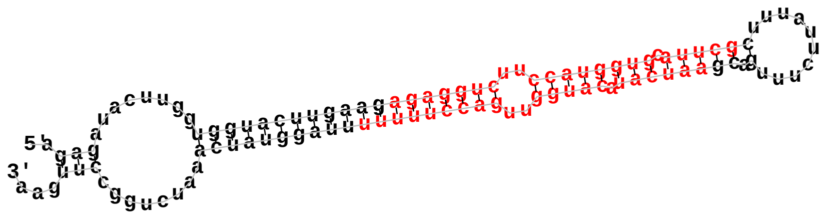

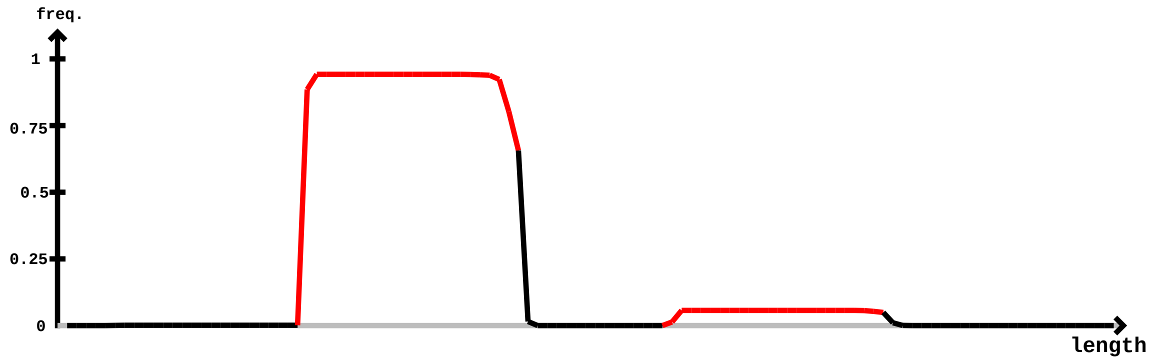


```
                                                                  oar-miR-154b-3p
                            oar-miR-154b-5p
5'- agagauacuuggugguacuugaagagaggucuuccauggugcauucgcuuuauucuuugacgaaucauacagguugaccuuuuuuuagguaucaaaucuggccuugaa   -3'    exp
    .(((........((((((((((((((((((..((((((((.((((((..........).))))))))).))))).)))))))))))))))))))........)))...   reads      mm         sample
    ....auacuuggugguacuugaag....................................................................................   1          0          seq
    .....uacuuggugguacuugaag....................................................................................   1          0          seq
    ........................agaggucuuccauggug...................................................................   1          0          seq
    ........................agaggucuuccauggugc..................................................................   1          0          seq
    ........................agaggucuuccauggugG..................................................................   1          1          seq
    ........................agaggucuuccauggugca.................................................................   3          0          seq
    ........................agaggucuuccauggugcau................................................................   26         0          seq
    ........................agaggucuuccauggugcauu...............................................................   202        0          seq
    ........................agaggucuuccauggugcauuc..............................................................   241        0          seq
    ........................agaggucuuccauggugcauucg.............................................................   1015       0          seq
    ........................agaggucuuccauggugcauucgc............................................................   23         0          seq
    .........................gaggucuuccauggugcau................................................................   1          0          seq
    .........................gaggucuuccauggugcauu...............................................................   3          0          seq
    .........................gaggucuuccauggugcauuc..............................................................   9          0          seq
    .........................gaggucuuccauggugcauucg.............................................................   81         0          seq
    .........................gaggucuuccauggugcauucgc............................................................   3          0          seq
    ..............................................................aaucauacagguugaccuu...........................   1          0          seq
    ..............................................................aaucauacagguugaccuuu..........................   4          0          seq
    ..............................................................aaucauacagguugaccuuuu.........................   5          0          seq
    ..............................................................aaucauacagguugaccuuuuu........................   12         0          seq
    ..............................................................aaucauacagguugaccuuuuuu.......................   1          0          seq
    ...............................................................aucauacagguugaccuuu..........................   1          0          seq
    ...............................................................aucauacagguugaccuuuu.........................   2          0          seq
    ...............................................................aucauacagguugaccuuuuu........................   54         0          seq
    ...............................................................aucauacagguugaccuuuuuu.......................   16         0          seq
    ...............................................................aucauacagguugaccuuuuuuu......................   1          0          seq
```
